# Legal and ethical implications of AI-based crowd analysis: the AI Act and beyond

**DOI:** 10.1007/s43681-024-00644-x

**Published:** 2025-01-07

**Authors:** Emmeke Veltmeijer, Charlotte Gerritsen

**Affiliations:** Department of Computer Science, Vrije Universiteit Amsterdam, De Boelelaan 1111, 1081 HV Amsterdam, The Netherlands

**Keywords:** AI-based crowd analysis, Ethical implications, AI Act, GDPR, Practical recommendations

## Abstract

The increasing global population and the consequent rise in crowded environments have amplified the risks of accidents and tragedies. This underscores the need for effective crowd management strategies, with Artificial Intelligence (AI) holding potential to complement traditional methods. While AI offers promise in analysing crowd dynamics and predicting escalations, its deployment raises significant ethical concerns, regarding privacy, bias, accuracy, and accountability. This paper investigates the legal and ethical implications of AI in automated crowd analysis, with a focus on the European perspective. We examine the effect of the GDPR and the recently accepted AI Act on the field. The study then delves into remaining concerns post-legislation and proposes recommendations for ethical deployment. Key findings highlight challenges in notifying individuals of data usage, protecting vulnerable groups, balancing privacy with safety, and mitigating biased outcomes. Recommendations advocate for non-invasive data collection methods, refraining from predicting and decision-making AI systems, contextual considerations, and individual responsibility. The recommendations offer a foundational framework for ethical AI deployment, with universal applicability to benefit citizens globally.

## Introduction

The global population has surged to more than 8 billion individuals [1], resulting in increased crowding in various locations. Unfortunately, densely populated areas are prone to accidents. Crowd tragedies happen on a yearly basis, with thousands of casualties in the last decade alone [2, 3]. Even small events and incidents, such as a goal during a sports game or an argument between people, can lead to serious escalation [4].

Efforts to control crowds and prevent such incidents include measures like placing fences, ensuring ample escape routes, managing traffic flow, and restricting visitor numbers [5]. However, these measures often fall short in adapting to dynamic situations. Artificial Intelligence (AI) emerges as a pivotal tool in this context. Thanks to advancements in processing power, data accessibility, and algorithm accuracy, intelligent systems can analyse crowd dynamics in real-time. AI algorithms capable of detecting emotions and behaviour, such as panic or aggression, offer valuable insights for crowd control and can aid in analysing crowd dynamics, predicting escalations, and recommending interventions. In a study conducted by Martella et al. [6], 10 crowd managers were interviewed about their work and practices. They shared that at that time AI-based technology played little to no part in their work, while they did consider AI technology to be an opportunity for future advancements in the field. While in recent years more technological advancements are being adopted for crowd management as AI algorithms keep improving both in accuracy and deployability, obtaining high performances when using these systems in an uncontrolled environment remains challenging [7, 8].

In recent years, a number of initiatives has been taken to deploy AI-based crowd analysis techniques to effectively manage crowds. In Saudi Arabia, the government has deployed AI technologies to be used during pilgrimages. Using smartphones and smart bracelets, efficiency is increased and people’s health is monitored, meant to increase the safety of visitors [9, 10]. Another example is Paris, where during the Olympic Games of 2024 cameras and drones were equipped with AI technology. This was used to optimise flow management and enable effective communication, but also to detect deviant and potentially dangerous behaviour to increase safety [11, 12].

While these technological advancements hold the potential to enhance crowd safety by enabling monitoring and early anomaly detection, the deployment of AI for behavioural and emotional crowd analysis raises significant ethical concerns. Specifically, four problematic themes can be identified in automated emotion detection: privacy, bias, accuracy, and accountability [13], which we posit are equally applicable to automated behaviour detection.

Cultural differences in emotional expression may lead to inaccuracies and biases in AI models, while automated decision-making raises questions of responsibility and therefore accountability [14–17]. These issues are reinforced when AI models have poor accuracy when deployed outside of controlled environments, risking misjudgements or even wrongful decisions that result in unfair treatment of certain individuals. Additionally, the collection of personal data for crowd analysis poses privacy risks. When people are monitored, their self-determination, individual autonomy, and right to consent can be undermined or violated [18, 19]. Worldwide, efforts are made to regulate the storage and use of personal data and AI systems, in order to protect these human rights [20–24]. The European Union (EU) has been the first to adopt a law for regulating AI, the Parliament approving it in early 2024 [25]. This law, the AI Act, describes what types of AI should be prohibited, and what types should meet additional requirements before being allowed enrollment. It is meant to complement existing EU law, and should specifically be seen next to the EU’s pre-existing data protection law, the GDPR.

In this paper, we aim to explore the legal and ethical implications of EU regulation on automated crowd analysis. The research question that this paper aims to answer is the following:

- To what extent do the AI Act and related legislation address the ethical concerns of bias, privacy, accuracy, and accountability in automated crowd analysis, and what recommendations can be made to stakeholders building and using these systems?

In order to answer this question, we will first discuss relevant regulations within the EU. After that, we examine what practical and ethical concerns remain after implementation of those regulations or are beyond their scope. We do this by considering the themes of privacy, bias and accuracy, and accountability. We then provide specific recommendations for future research and implementation. The framework adopted for this study is therefore a regulatory analysis with ethical evaluation, focusing on the intersection of law, technology, and ethics. This approach is suitable for addressing the research question, as it allows for a critical assessment of current regulatory efforts and offers constructive recommendations grounded in both legal and ethical considerations.

## Legislation and its implications

With both possibilities and risks rising with the development of new techniques and applications, the call for regulations regarding AI technologies and data in general is ever increasing. Guidelines, proposals and laws have seen the light in recent years, on both national and international levels [26]. This section aims to provide a comprehensive overview of the European Union’s efforts towards unified regulations.

In 2016, the EU adopted Regulation (EU) 2016/679, better known as the General Data Protection Regulation (GDPR),^1^ which took effect in 2018. This regulation aims to protect the fundamental rights of EU inhabitants regarding handling of personal data. The processing of data for law enforcement falls outside the scope of this regulation, and is instead included in the Law Enforcement Directive, or Directive (EU) 2016/680 (Chapter I(19)).^2^ While this regulation was an important step for legislation keeping up with a changing society, it did not account for the field of AI and the rapid developments therein.

In May 2024, the European Council formally adopted the Artificial Intelligence Act (AI Act) [27]. Its goal is to protect ‘health, safety, fundamental rights’, while ensuring that the EU can remain competitive when it comes to technological innovations, and ensuring that its inhabitants can safely reap these technologies’ benefits. These fundamental rights include, amongst others, ‘the right to human dignity, respect for private and family life, protection of personal data, freedom of expression and information, freedom of assembly and of association, and non-discrimination, [...], right of defence and the presumption of innocence’ (AI Act, Chapter I(28a)). It is intended to serve as a complement to the GDPR and the Law Enforcement Directive. Acknowledged are the advantages AI can bring to society as a whole, but also the risks of causing material or immaterial harm to the rights protected by EU law (AI Act, Chapter I(3) and I(4)). It is grounded on the EU’s seven principles for trustworthy AI (human agency and oversight; technical robustness and safety; privacy and data governance; transparency; diversity, non-discrimination and fairness; societal and environmental well-being and accountability) (AI Act, Chapter I(27)). While this paper is structured following ethical themes that have specifically been found to be problematic in automated emotion detection (privacy; bias; accuracy; an accountability), these seven principles will be discussed implicitly as they are part of the AI Act.

### Scope of GDPR

The rules of data protection as laid down in the GDPR are only applicable to information that can be used to identify a natural person, i.e., personal data. Information not related to a person as such, or anonymised data, is therefore not bound to this regulation (GDPR, Chapter I(26)). Natural persons should be made aware if and how their data will be used (GDPR, Chapter I(39)). Additionally, natural persons have ‘the right not to be subject to a decision based solely on automated processing’ where the decision significantly affects the person subjected to it (GDPR, Article 22(1)). Analysing a crowd by using identifiable data of individuals within the crowd, such as speech recordings or images, must therefore adhere to the rules set by the GDPR. The practicality of notifying individuals about their data being collected could become challenging or even impossible, especially when analysing large groups, depending on the analysis environment.

The GDPR emphasises the importance of processing personal data lawfully, indicating that such processing may be allowed if it serves a public interest or is necessary for the performance of a task carried out in the public interest (GDPR, Art. 5(1), Art. 6(1)). Determining what constitutes public interest lacks an exhaustive list of requirements, but references include public health, humanitarian purposes, and, according to the AI Act, the protection and improvement of the environment (AI Act, Art. 54(1)). When considering any approach to crowd analysis, it becomes crucial to assess whether the purpose aligns with public interest, such as enhancing event security for the well-being of attendees. Nevertheless, any use of AI in this context should involve a careful evaluation of the rights of the individuals involved.

Article 9(2) of the GDPR describes processing of the following personal data as prohibited: ‘personal data revealing racial or ethnic origin, political opinions, religious or philosophical beliefs, or trade union membership, and the processing of genetic data, biometric data for the purpose of uniquely identifying a natural person, data concerning health or data concerning a natural person’s sex life or sexual orientation’. In the context of crowd management, a system’s main goal might not be to categorise people as such, but personal data revealing for example ethnic origin or political opinions could be collected nonetheless, particularly in protesting crowds where the nature of the crowd might be indicative of such personal information. However, Article 9(2) lists several exceptions, among which ‘processing relates to personal data which are manifestly made public by the data subject’ and when there is significant public interest (while continuously requiring respect of fundamental rights of the natural person). Apart from the point of public interest, as was discussed before, one could claim that for example people participating in a political demonstration are making their views publicly known, which would mean that collecting such personal data would not be prohibited per se, based on the GDPR alone. However, one should keep these people’s intentions in mind: declaring something on a social medium, where it is available to all, may be different from participating in a protest (or even simply being present during a protest) which happens offline and locally.

Both the GDPR and the AI Act mention children, as deserving of extra protection (GDPR, Chapter I(38)) and holding specific rights that require additional consideration when implementing a (high-risk) AI system (AI Act, Chapter I(28a) and Art. 9(8)). The AI Act additionally mentions other vulnerable groups of people to deserve the same consideration. For general crowds, unless those at events with age restrictions, it is not possible to state with certainty whether all people under analysis are 16 years or above. The rights reserved for these people would therefore be hard, if not impossible, to do justice with an increasing crowd size.

### Scope of AI Act

Before analysing the consequences the AI Act will have for the field of automated crowd analysis and crowd management, it is important to highlight the exceptional position reserved for science. It states: ‘This Regulation should support innovation, should respect freedom of science, and should not undermine research and development activity. It is therefore necessary to exclude from its scope AI systems and models specifically developed and put into service for the sole purpose of scientific research and development.’ (AI Act, Chapter I(25)). This exclusion holds until AI systems are ‘put into service or placed in the market’, and acknowledges that any research performed should adhere to ethical and professional standards for scientific research, as well as applicable EU laws. For the remainder of this section, we will discuss the implications of the AI Act on systems that fall outside this exception.

Within the proposed AI Act, AI algorithms are divided into four categories. The first are those carrying unacceptable risk, which should be prohibited. The second category is that of high-risk algorithms, which should meet specific requirements. The third category, limited risk, should adhere to transparency obligations. The final category contains algorithms carrying minimal or no risk, that can be employed freely.

Taking a closer look to what kind of algorithms fall into each category, we find that ‘real-time’ remote biometric identification for law enforcement is prohibited (AI Act, Art. 5(1)). There are three exceptions to this rule, in which biometric identification for law enforcement may be used in real-time under strict conditions (AI Act, Art. 5(1)). Biometric data is defined as ‘personal data resulting from specific technical processing relating to the physical, physiological or behavioural characteristics of a natural person, which allow or confirm the unique identification of that natural person, such as facial images or dactyloscopic data’ (GDPR, Art. 4(14) and AI Act, Art. 3(33)), the AI Act furthermore stating that biometric data ‘can allow for the authentication, identification or categorisation of natural persons and for the recognition of emotions of natural persons’ (AI Act, Chapter I(7)). Additionally, emotion recognition systems to be used in the workplace or in education are to be prohibited as well, given the ‘limited reliability, the lack of specificity and the limited generalizability’ of such systems (AI Act, Chapter I(26c), Article 5(1)). Aligning with the presumption of innocence, people should furthermore ‘always be judged on their actual behaviour’, and never on behaviour predicted by AI (AI Act, Chapter I(26a)). Therefore, additionally to be prohibited are AI systems that are used to predict if someone will commit a crime, ‘based solely on the profiling of a natural person or on assessing their personality traits and characteristics’ (AI Act, Art. 5(1)). Examples or characteristics given are, among others, nationality, number of children, and debt (AI Act, Chapter I(26a)). While expressed emotion (e.g., an angry expression) or physical movement (e.g., a raised fist) are not explicitly mentioned and might not be considered as characteristics, it should be clear that predicting whether something will happen rather than detecting when something is actually happening is something to be very wary of and to consider carefully. This should avoid or limit biases present in such systems, which could coincide with increased accuracy.

Considered to be high-risk are algorithms for biometric categorisation ‘according to sensitive or protected attributes or characteristics based on the inference of those attributes or characteristics’, and systems for emotion recognition used outside the working place and education (AI Act, Annex III(1)). An emotion recognition system is defined as ‘an AI system for the purpose of identifying or inferring emotions or intentions of natural persons on the basis of their biometric data’ (AI Act, Article 3(34)), referring to emotions such as happiness, sadness, and anger, but not including ‘the mere detection of readily apparent expressions, gestures or movements, unless they are used for identifying or inferring emotions’ (AI Act, Chapter I(8a)). The definition of biometric categorisation, as a system intended to assign individuals to ‘specific categories on the basis of their biometric data’ (AI Act, Article 3(35)), leads to some unclarity. These categories can relate to, amongst others, ‘behavioural or personality traits (AI Act, Chapter I(7b)). Many machine learning models have classification of some sort as their goal, which by definition would mean the categorisation of people, if features of these people are used as input data. Categories within a crowd could for example be fighting people and non-fighting people, or violence and non-violence. Such a classification would then be considered high-risk when the analysis is done on an individual level requiring personal data, even if no personal data is stored.

High-risk models should have a level of transparency for their users, in order for them to understand the model’s output and know how to work with it (AI Act, Article 13(1)). Specifically, people exposed to a system for emotion recognition or biometric categorisation should be informed of the operation of the system (AI Act, Article 52(2)). Furthermore, human oversight should be present when using high-risk systems (Article 14(1)). This user should be able ‘to decide, in any particular situation, not to use the high-risk AI system or otherwise disregard, override or reverse the output of the high-risk AI system’ (Article 14(4)). This relates to the issue of accountability, by ensuring that humans will keep the final say. Furthermore, having a human in the loop who keeps oversight and can intervene, could reduce the effect of any biases in the system.

The AI Act specifically mentions the importance and necessity of protecting privacy, minimising data, and ‘data protection by design and by default’ (AI Act, Chapter I(45a)). Specific responsibility is furthermore assigned to the developers of AI systems, to design and develop systems that ‘achieve an appropriate level of accuracy, robustness, and cybersecurity’ (AI Act, Article 15(1)). Additionally acknowledged is the role of those employing the developed system: they should understand how the system works and remain critical of potential risks associated with its use (AI Act, Chapter I(58b)).

The rules described in the AI Act apply only in publicly accessible spaces. These are defined as locations the public can access, regardless of if they are privately owned or publicly owned. This means that if a certain location has conditions for entering it, such as requiring an entry ticket, the Regulation still considers it a public space (AI Act, Art. 3(39)). Considering the Act applies to publicly accessible spaces, and its definition of that, it can safely be stated that the AI Act applies to most, if not all, systems intended for crowd analysis, as these are unlikely to take place in the privacy of one’s own home.

### Summary of GDPR and AI Act implications

In conclusion, the European Union has laid a foundation for the regulation of AI systems and data protection through instruments such as the GDPR and the proposed AI Act. The GDPR sets forth fundamental principles for the processing of personal data, ensuring individuals’ rights and privacy. The AI Act categorises algorithms by risk and imposes transparency and oversight requirements.

Considering the EU regulations in light of crowd analysis, there are several conclusions to be drawn. For the remainder of this section, we are assuming data collection for purposes other than law enforcement. The collection of individual’s personal data then falls under the GDPR, encompassing all crowd analysis systems that work with an individual-based approach. Lawful processing, justifiable by public interest, is crucial, with added precautions for children’s data. Furthermore, people should not be judged based on predicted behaviour, but on their actual behaviour: therefore, systems should be designed to detect rather than to predict. Collecting data that allows identification of individuals is additionally considered high-risk per the current AI Act. These facts combined tell us that collecting data about individuals for crowd analysis is possible, but the system and its users should adhere to particular restrictions and requirements.

The AI Act applies to all publicly accessible spaces, impacting all AI-based crowd analysis systems. According to the act, emotion prediction as well as algorithms assessing characteristics of individuals or groups, are considered high-risk. Depending on the definition of ‘characteristics’, this categorisation may be inherent to any form of automated crowd analysis. Finally, the AI Act mandates transparency and human oversight for high-risk models. Integrating these principles into crowd analysis systems at an early stage of development is crucial.

## Remaining concerns and suggested good practices

In this section, we will consider the themes of privacy, bias and accuracy, and accountability. For each theme, we analyse practical and ethical concerns that arise from the discussed legislation or are not addressed by it. Based on this, we suggest solutions and good practices.

### Privacy

Privacy, and the protection thereof, is considered a human right and one of the main objectives of personal data protection, as embedded in both the GDPR and the AI Act and discussed in Sect. 2. Privacy is important for many reasons, including protection of one’s interests or integrity, having control over what information is shared with whom, but also allowing for diverse social relationships with various people [28]. In the context of automated crowd analysis specifically, monitoring people can deprecate their self-determination, individual autonomy, and consent [18, 19].

***Identifying concerns*** From the GDPR and the AI Act, two practical concerns are identified. The first is that individuals should be made aware if and how their data will be used, the second is the extra protection required for personal data of children and other vulnerable groups. Both of these rules become increasingly impractical when the size of the crowd under analysis increases: when analysing a crowd of hundreds or even thousands of people (without requiring an entry ticket or age limit), it might not be feasible to notify all people of the way their data will be used. Putting up signs that indicate the collection of data would not be enough, as it would not be guaranteed that every member of that crowd is able to see and understand that information. Furthermore, one cannot be certain that there are no minors within the crowd whose personal data would unknowingly be collected.

Assuming that the goal of automated crowd analysis is to benefit those under analysis, we argue that the effect the analysis has on the crowd determines whether performing the analysis is ethically just. Following this perspective, it is the outcome of an action which determines if the action was ethically ‘just’ or not. The factors indicating the importance of privacy mentioned before, such as retaining individual autonomy, self-determination, and maintaining diverse social relationships, are beneficial to an individual. From that perspective, preserving privacy would be worthwhile, as it would increase the ‘happiness’ of the crowd. One could argue that the intended effect of such crowd analysis, increased safety, would also be beneficial to the individuals in the crowd. However, if this is done while harming one’s privacy, there are two interests that counter each other. This is an ethical concern that remains after implementation of the AI Act: such data collection is permitted under certain circumstances, for instance when it serves public interest. In these cases and with regular crowd analysis systems, the protection of safety would always be at odds with the protection of privacy.

***Recommendations*** We have identified three concerns that regard privacy. First, that with large crowds in public, it would be unfeasible to notify every individual of their data being collected. Second, that for such large crowds it could not be guaranteed that no personal data of underaged individuals would be collected. Third, that protecting people’s safety through automated crowd analysis would always come at a loss of privacy. To tackle these issues, we believe that the way forward is to work on systems that analyse crowd-level data instead of combining individual data. Such methods work with crowd-level features, that do not require processing of any personal data. Concrete examples include studies working with frame-wide motion features from videos, or with features from crowd audio in which no individual voices can be identified [29–31]. By not processing personal data, such an analysis would not violate privacy rights, meaning that the advantage of increased physical safety would no longer come at a loss of privacy. An overview of the concerns and recommendations discussed in this section can be found in Table 1.

**Table 1:** Overview of privacy-related legal requirements, concerns, and recommendations

Legal requirements	Concerns	Recommendation
1. Individuals should be made aware if and how their data will be used 2. Personal data of children and other vulnerable groups is deserving of extra protection	1. In large crowds, not every individual can be informed 2. In large crowds, one cannot be certain about the age of each individual 3. The protection of safety is at odds with the protection of privacy	1. Work on systems that analyse crowd-level features, without processing any personal data

### Bias and accuracy

For any AI system, it is of the utmost importance that models show a robust performance when tested and employed in real-world environments. The AI Act underlines this, stating that accuracy, reliability, and transparency are crucial to ‘avoid adverse impacts, retain public trust and ensure accountability and effective redress’ (AI Act, Chapter I(59)). Therefore, high-risk systems are required to meet an appropriate level of accuracy and robustness (AI Act, Chapter I(74)). Inaccurately trained AI systems can lead to unfair and biased outcomes. Such biases may ‘have a negative impact on fundamental rights or lead to discrimination prohibited under Union law’ (AI Act, Chapter I(67)), and should be mitigated during development stages. Otherwise, in the case of automated crowd analysis, wrongful output could result in unfair treatment of certain individuals based on prejudiced or otherwise inaccurate assumptions. Since biased results may be an indicator of an inaccurate AI system, the themes of bias and accuracy will be treated together in this section.

***Identifying concerns*** According to the AI Act, as discussed in Sect. 2, people should be judged on their actual behaviour and not on predicted behaviour. Specifically prohibited are AI systems that predict if someone will commit a crime, based on the profiling of that person or assessing their personality traits and characteristics. The examples given do not include expressed emotions or physical movements, which seems to leave room to use such features for prediction as they may not be considered characteristics of that person. Furthermore, when the prediction is about something other than the likelihood of someone committing a crime, it may also not be prohibited. This raises ethical concerns, as the biased outcomes or wrongful decisions such a system could lead to are therefore not fully covered by the AI Act. While not all inaccurate predictions may be harmful, the prediction of behaviour will generally be more likely to display biases than the detection of it, the former having to base itself on features not directly relating to the behaviour itself.

***Recommendations*** Addressing the concern of biased outcomes and wrongful decisions of a predictive system, we argue that focus should lie on detecting particular behaviour as early on as possible rather than predicting it. Intervention might then still be possible, while limiting biases and thereby increasing accuracy. A different approach could be to detect particular expressions that may, or may not, lead to a particular behaviour. To provide an example, a system could detect angry people, because anger may lead to aggression. When the anger detection reaches a robust performance, this would be a ‘fairer’ system in the sense that it would treat everyone the same by considering their current expression. However, a system meant to directly predict aggression would be a different story, as it may base its prediction on biased or otherwise incorrect features.

While the importance of a decently trained, accurate, and robust system might be clear, the question of when a system meets the ‘appropriate level of accuracy’ required by the AI Act is not as straightforward. A system scoring perfectly may not be realistic, and the accuracies reached by state-of-the-art AI models highly differ per field, application, and testing condition. This is why we argue that context-awareness and understanding the need of those under analysis are important in automated crowd analysis. Therefore, our second recommendation for tackling bias and accuracy problems is to consider the context, which means that there is not a ‘one size fits all’ solution for automated crowd analysis. Instead, depending on contextual factors such as the type of gathering, cultural background of the crowd, and expected results, the AI system deployed should be adjusted or revised to fit the needs of those subjected to it. What is ethical and the ‘right’ thing to do will depend on many different aspects, including but not limited to the type of event and type of attendees such as their interpersonal relationships, region and culture, legislation, and expected results.

The third recommendation within this theme relates to the second one. Given the importance of context for crowd analysis, crowd managers should consider the type of attendees that make up the crowd. A crowd manager should first and foremost think of using such a system for improving the attendee’s experience and safety above all else. But by truly trying to understand the attendee’s reality, they must ask themselves the following questions: will the attendees actually benefit from the system? Are the attendees able to understand the risks, both of attending with and without the said system, and form an educated opinion on them? Are the attendees vulnerable, safety-wise or privacy-wise? Attendees should be able to understand the ‘protection’ they are receiving, in this case the automated system for crowd emotion and behaviour detection analysing them, in order to form an opinion. To enhance understanding among attendees while also being open to their response to the protection provided, examples of possible solutions are to send out questionnaires asking about people’s experience (before, during, or after an event), or have employees walk the field to provide information, gather opinions and perceive people’s immediate response. It is important to note that this may lead to different outcomes, given different groups of people with changing context, circling back to the contextual component. An overview of the concerns and recommendations discussed in this section can be found in Table 2.

**Table 2:** Overview of bias and accuracy-related legal requirements, concerns, and recommendations

Legal requirement	Concern	Recommendations
1. No crime prediction based on individual profiling or assessing personality traits and characteristics	1. Prediction based on emotions/movements or non-crime prediction, and therefore potentially harmful and biased predictions, are still possible	1. Detect rather than predict 2. Consider the context, no ‘one size fits all’ 3. Understand the crowd, and ensure they understand the analysis

### Accountability

The theme of accountability relates to the question of who is to be held accountable for the employment of automated crowd analysis and its consequences. When there is a human in the loop, that person would be the one responsible for the final decision to act. However, in the case of automated emotion and behaviour recognition for crowd management, a decision to intervene may in principle be taken without any human intervention. This may be based on wrongful or biased results, and lead to unfair treatment or even harming of those subjected to it. Even if a human is the one to take the final call to act based on the outcome of an AI system, the question remains who is accountable for the effect it has, to what extent the people involved hold accountability, and what should be done by those accountable to prevent wrongful doing. Following the AI Act, humans should be kept in the loop at all times when deploying high-risk systems, where the user should be able to opt out of using the system, or to ‘disregard, override or reverse’ its output (AI Act, Article 14(1) and Article 14(4)).

***Identifying concerns*** Even when a human is kept in the loop and is able to choose not to work with the AI system’s output, there are certain dangers and concerns associated with accountability. When a human is tasked with checking the AI system’s output to avoid wrongful doings, mistakes or oversights can happen. When the AI system is merely detecting a particular emotional expression or behaviour, providing the user with knowledge, the consequences of overlooking something might be small. For example, a system built to assist in crowd surveillance might give a notification if it detects an act of violence. This will not lead to any action just yet, but will draw the attention of the security guard monitoring the camera feeds to the location of spotted violence. The security guard can then see for themselves if this is indeed the case, and decide if and how intervention should take place [32]. The number of steps required before any action is taken will decrease the chance of mistakes or wrongful actions taking place. However, when the system itself decides on an action to take, based on whether or not something was detected, it requires security personnel only to approve or disapprove. As a result, mistakes are more likely to occur, which could potentially lead to more adverse effects.

***Recommendations*** We have identified the concern of decision-making AI systems that remain legal to some extent. Since AI systems forming or suggesting decisions on their own are more prone to errors, we argue to take it a step further than required by the AI Act, and avoid decision-making AI systems for crowd analysis altogether. While we acknowledge that AI systems can help provide suggestions on what actions to take, we believe that it should be left to the human in the loop to decide on the necessary actions, based on information provided by the AI system. As indicated in the concrete example provided before, this could be done by bringing the attention of security personnel to particular areas or groups of people, without assigning an action to this.

Furthermore, we want to substantiate the importance of not simply following the rules because one ‘has to’, but to have the other’s best interest in mind. People are to be treated as ends by themselves, and not merely means to an end [33]. This means that with automated analysis of crowd emotion and behaviour for enhancing public safety, people are analysed to reach that goal, making them the means. However, the goal itself is to enhance the safety of the public, making them the end as well. They are part of the methodology deemed necessary or at least helpful in order to serve them. The intention is for it to be in the best interest of those observed, not those observing. We stress that it is up to those involved in automated crowd analysis to take responsibility in aligning their actions with what is best for those subjected to the analysis, even if that means taking things further than required by the AI Act or other legislation. 

Considering automated crowd analysis, there are multiple people or groups of people involved in the process of putting such an automated system into practice: the person in charge of crowd management, whose choice it is whether or not to use such a system, the developer of such a system when employed commercially, and the scientist experimenting with such systems and their performances or effects. The objective of the crowd manager (amongst others) is to protect the attendees as much as possible, keeping them safe both physically and privacy-wise. This means that the ‘right’ thing to do would be to perform the action that keeps them safest. The developer’s objective will be to build a safe and functioning system. While their main priority might be to have the application meet the requirements in terms of functionality, meeting requirements on storage of data, data security, and transparency where needed are all the more important. These requirements are influenced by multiple parties: (governmental) regulations on the one side, the party commissioning the project on the other side, and then the developer in the middle, meaning that they will not possess full power and flexibility to make all design choices. However, it is still their responsibility to not only adhere to regulations and requested requirements, but also to their own moral judgement of what a good system ought to do, taking into account not just the functionality but also the potential uses and misuses of such a system. This is not an objective guideline, as what is morally right to one person may not be so to another person. However, the intention is to encourage those involved in these processes to think about and discuss ethicality, in addition to adhering to the regulations already in place.

**Table 3:** Overview of accountability-related legal requirements, concerns, and recommendations

Legal requirement	Concern	Recommendations
1. Keep humans in the loop for high-risk systems and enable them to override the system’s output	1. Having a system that decides on what action to take, and asks the user only to verify, is prone to errors and can lead to adverse effects	1. Have the system provide the user with information based on which the user must decide what to do, no decision-making systems 2. Take responsibility in aligning one’s actions with what is best for those subjected to the analysis 3. Aim for scientists to adhere to the AI Act as much as possible

Looking at the role of scientists, we saw that the AI Act excludes from its scope AI systems for scientific research and development (AI Act, Chapter I(25)). While this leaves a lot of freedom for research to be done on automated crowd analysis, we argue that ‘just because you can, does not mean you should’. The question is what the purpose of such research would be: if adopting developed systems, or future versions of them, to real-world scenarios is prohibited, it should be carefully considered what the relevance of such a line of research would be. There may be situations in which research of automated crowd analysis could lead to findings that would ultimately benefit society or science in other respects. Moreover, it is important to keep up with technological knowledge and skills developed globally. We therefore do not mean to say that all research in this field should be brought to an end, but we believe that research on automated crowd analysis should aim to adhere to the regulations intended for non-scientific systems to the extent possible and thereby take on a leading role within the EU. We plead that every party involved in automated crowd analysis should take responsibility for their own part, with a special role for scientists given the freedoms assigned to them by the AI Act. An overview of the concerns and recommendations discussed in this section can be found in Table 3.

## Conclusion

New methodologies are being developed for automated crowd analysis, leading to increased application possibilities as well as increased debate on the ethicality of such systems. Specifically, four problematic themes can be identified [13]: privacy, bias, accuracy, and accountability. To shed light on this complex matter, we reviewed current legislation, focusing on the European Union’s GDPR and AI Act. Our analysis indicates that automated crowd analysis is permissible under specific conditions and restrictions. We then explored concerns that remain after implementation of the AI Act, by discussing the main themes of privacy, bias and accuracy, and accountability. For each theme, we discussed the relevance, remaining concerns, and recommendations for mitigating these concerns.

The research question holds: to what extent do the AI Act and related legislation address the ethical concerns of bias, privacy, accuracy, and accountability in automated crowd analysis, and what recommendations can be made to stakeholders building and using these systems?

***Concerns*** Based on the disquisition discussed throughout this paper, the authors found the following concerns to remain:

1. Notifying individuals of how their data will be used (as required per the GDPR) becomes infeasible in large crowds in public spaces;
2. Providing extra protection to personal data of children and other vulnerable groups or guaranteeing that their data will not be collected (as required per the GDPR and AI Act), becomes impossible in large crowds that did not provide personal information (such as age) beforehand;
3. Protecting physical safety through automated crowd analysis comes at a loss of protecting privacy, and vice versa;
4. Predicting behaviour remains possible, depending on the features used and the type of behaviour that is predicted, leaving room for biased outcomes or wrongful decisions;
5. AI systems can make or recommend decisions, as long as there is a human in the loop in the case of high-risk systems, increasing the chance of making mistakes that could have harmful effects on those under analysis.

***Recommendations*** Given these concerns, we discussed possible solutions and considerations. Specifically, the following recommendations were made:

1. Work on systems that analyse crowd-level data, that do not require the collection of personal data (mitigating concerns 1, 2, and 3);
2. Work on systems that detect emotions or behaviour, rather than predicting them (mitigating concern 4);
3. Work on systems that do not make or suggest decisions, but instead provide the human in the loop with information based on which they can decide what action to take themselves (mitigating concern 5);
4. When deciding on if and what kind of automated crowd analysis should be performed, consider contextual factors such as interpersonal relationships, region and culture, and expected results;
5. Ensure that those providing the analysis and those under analysis understand each other: those analysing should ensure that it is in the best interest of those under analysis, while those under analysis should be fully informed about the analysis taking place, and should be able to respond and share their opinion with the one analysing;
6. Every party involved in automated crowd analysis should take their own responsibility to not only adhere to legislation, but to furthermore think about and discuss their own moral views and keeping in mind what the ultimate goal (safety of people analysed) should be;
7. Scientists, although given more freedom in the AI Act, should aim to adhere to the regulations intended for non-scientific systems to the extent possible, considering the ultimate intended societal relevance of their research if they do not.

In conclusion, we argue that automated crowd analysis should only be done under strict conditions, and never be a goal in itself: rather, it should be a method to serve the goal of increasing safety and serving public interest. Next to the fact that such a system should adhere to laws and regulations, it should always keep human’s autonomy intact: everyone should at least be aware of what is happening with their data, and should be able to choose whether or not they want to be a part of that (apart from analysis for law enforcement purposes). The recommendations provided should be seen a starting point of the important discussion on if and how we as a community should work on automated crowd analysis.

***Discussion*** It should be taken into account that this work focuses on EU regulations and its consequences within the EU. However, it does not explicitly discuss the effect that developments in other parts of the world will have on the EU. While all systems deployed within the EU should adhere to these regulations, regardless of their origin, scientific and commercial advancements made elsewhere will undoubtedly have an effect on the European market and scientific community. Further work should carefully review the potential effects of such global developments, and how to ensure that the EU can remain competitive, both commercially and scientifically, while still adhering to the regulations in place and following ethical recommendations such as those laid out here. Furthermore, the authors want to emphasise that while the current work has focused on AI legislation within the EU, similar regulations are being and have been developed worldwide. For comparative analyses of the AI governance in different global regions we refer the reader to Mökander et al. [34], Roberts et al. [35], and for an overview of global AI and data regulations we refer the reader to Jones et al. [21].

We stress that the concerns raised and recommendations made are based on our own interpretation, and may contain biases themselves. However, they can, and we believe they should, serve as a framework in which everyone should take their own responsibility to act ethically. In order to achieve this, everyone involved in any part of such a system, should remain critical to not only the system itself, but also their own moral beliefs and actions. Field-specific guidelines, campaigns and education might be of help to raise awareness and motivate people to think about what they consider to be ethical, and what they can do to align their actions with those beliefs.

Finally, while this disquisition of practical and ethical concerns and the recommendations that followed from them are based on EU regulations, the authors believe that they are universal. The aim of these recommendations is to respect each individual’s privacy, rights, and interests, while serving public interest. As such, these goals could be beneficial to citizens everywhere.

## Footnotes

1 Consolidated text (2016-05-04): Regulation (EU) 2016/679 of the European Parliament and of the Council of 27 April 2016 on the protection of natural persons with regard to the processing of personal data and on the free movement of such data, and repealing Directive 95/46/EC (General Data Protection Regulation) [2016] OJ L 119/1.

2 Consolidated text (2016-05-04): Directive (EU) 2016/680 of the European Parliament and of the Council of 27 April 2016 on the protection of natural persons with regard to the processing of personal data by competent authorities for the purposes of the prevention, investigation, detection or prosecution of criminal offences or the execution of criminal penalties, and on the free movement of such data, and repealing Council Framework Decision 2008/977/JHA [2016] OJ L 119/89.
